# Diabetes and Bone Involvement in Primary Hyperparathyroidism: Literature Review and Our Personal Experience

**DOI:** 10.3389/fendo.2021.665984

**Published:** 2021-04-19

**Authors:** Elena Castellano, Roberto Attanasio, Alberto Boriano, Valentina Borretta, Francesco Tassone, Giorgio Borretta

**Affiliations:** ^1^ Department of Endocrinology, Diabetes and Metabolism, Santa Croce and Carle Hospital, Cuneo, Italy; ^2^ IRCCS Orthopedic Institute Galeazzi, Endocrinology Service, Milan, Italy; ^3^ Medical Physics Department, Santa Croce and Carle Hospital, Cuneo, Italy

**Keywords:** primary hyperparathyroidism, diabetes therapy, bone, diabetes mellitus, diabetes drugs

## Abstract

**Background:**

Primary hyperparathyroidism (PHPT) and type 2 diabetes mellitus (T2DM) are common endocrine disorders impacting on skeletal health, whose concomitant occurrence is becoming more frequent.

**Patients and Methods:**

We searched the PubMed database from the National Library of Medicine about the relationship between T2DM and its treatment and bone manifestations of PHPT. Thereafter, we retrospectively evaluated a consecutive series of 472 PHPT patients. Among them 55 were also affected by T2DM. At the diagnosis of PHPT we compared bone turnover markers and bone densitometry between 55 patients with and 417 without T2DM and in the former group according to antidiabetic treatment.

**Results:**

Few data are available about T2DM and PHPT bone involvement, studies about T2DM treatments and PHPT bone manifestations are lacking. Among patients with PHPT of our series, those with T2DM were older, had a lower prevalence of osteitis fibrosa cystica, higher lumbar and femoral T-scores than the remaining patients. No difference was disclosed among the diabetic patients according to ongoing antidiabetic treatment, even though modern treatments were under-represented.

**Conclusions:**

No clinical study specifically evaluated the impact of T2DM on bone involvement in PHPT. In our experience, diabetic patients resulted more frequently “mild asymptomatic” than non-diabetic patients and showed a lower prevalence of radiological PHPT bone manifestations. The treatment of T2DM does not seem to affect the biochemical or clinical features of PHPT in our series. Further studies are needed to fully disclose the influence of T2DM and antidiabetic treatment on bone health in patients with PHPT.

## Introduction

Primary hyperparathyroidism (PHPT) is a common endocrine disorder, nowadays usually diagnosed at the pauci-asymptomatic stage (1). Main target organs are bone and kidney, but symptomatic PHPT has been also associated with an increased prevalence of cardiovascular (CV) and metabolic abnormalities (2), in particular insulin-resistance (IR) (3), type 2 diabetes mellitus (T2DM) (4) and dyslipidemia (5), in turn related to a higher CV mortality risk than in the general population (6).

While the beneficial effect of parathyroidectomy (PTX) on bone and renal involvement is widely demonstrated (1), only limited and controversial data still exist about the CV manifestations in patients with asymptomatic PHPT (aPHPT), before and after PTX (2, 6). The presence of T2DM and CV involvement are not actually included among the criteria for surgery recommended by latest guidelines for aPHPT management (7).

An increased fracture risk, particularly at the hip, is a recognized complication of T2DM (8), even if bone mineral density (BMD) is not reportedly impaired and the factors underlying increased fracture risk have not yet been fully established.

Moreover, drugs used in the treatment of T2DM may have both direct and indirect effects on bone health and fracture risk (8). In particular, fracture risk is increased by thiazolidinediones (TZD), likely through the inhibition of osteoblastogenesis in both sexes (9), and perhaps by canagliflozin, an inhibitor of sodium-glucose transporter 2 (SGLT-2) (10, 11). On the other hand, dipeptidyl-peptidase 4 (DPP-4) inhibitors seem to exert a protective effect on bone (12).

Our review on literature data evidenced a lack of clinical study evaluating the impact of T2DM and its treatments on bone involvement in PHPT. The influence of T2DM and T2DM therapies on bone involvement in PHPT, if any, is still largely unknown. We have thus evaluated, in a large monocentric series of PHPT, the impact of T2DM and of antidiabetic treatments on PHPT bone manifestation.

## Patients and Methods

### Design

Firstly, we performed a review of clinical series and meta-analyses about the relationship between T2DM, its treatments and bone manifestations of PHPT.

Thereafter, a retrospective survey was conducted on the medical records of all patients diagnosed with PHPT who attended our department between January 1997 and December 2020.

The study was conducted in accordance with the Declaration of Helsinki and was approved by the Institutional Review Board and the Ethical Committee of our institution. No informed consent was required for this study because we only retrospectively accessed a de-identified database for analysis purposes. All data were collected as part of routine clinical and psychological procedures.

### Patients

The patients were referred by general practitioners, primary care clinics, and subspecialty clinics. PHPT diagnoses had been established by the presence of hypercalcemia and concomitant inappropriately raised serum PTH levels on at least two separate occasions (reference range for calcium levels 8.4–10.2 mg/dL and for PTH <65 ng/L, for details see below).

Patients diagnosed with MEN, hyperparathyroidism-jaw tumor syndrome, familial hypocalciuric hypercalcemia, and parathyroid carcinoma were excluded.

No patients had been taking calcium or vitamin D supplements, estrogens or testosterone or selective estrogen receptor modulators, or bone-active medications for at least six months.

In agreement with Bilezikian et al. (7), patients were classified as asymptomatic PHPT according to a lack of radiological signs of bone involvement, nephrolithiasis, and symptoms of hypercalcemia. Regarding bone involvement, all patients had routinely undergone dual X-ray absorptiometry (DXA) and a radiographic evaluation of the skull and hands to check for signs of excess PTH effects on bone, such as osteitis fibrosa cystica (OFC), subperiosteal resorption in fingers, salt and pepper mottling of the skull, and brown tumors. Patients with fragility fracture were considered symptomatic for bone involvement. In terms of kidney involvement, patients were classified as symptomatic either if they had a recorded positive history for renal stones (ultrasound examination, urography, plain radiography, history of passing stones, or their endoscopic or surgical removal), or if renal stones (or calcinosis) had been diagnosed by routinely performed ultrasound in either asymptomatic or symptomatic patients at physical examinations.

Asymptomatic patients who do not meet the surgical criteria set out by the updated international guidelines (12), where considered “mild asymptomatic” PHPT (13).

Diagnoses of T2DM were based on the American Diabetes Association criteria (14). T2DM duration (from time of the diagnosis) was extracted by medical records.

### Methods

We searched the PubMed database from the National Library of Medicine by using the following keywords: ‘Diabetes mellitus’, ‘T2DM’, ‘diabetes therapy’, ‘diabetes medication’, ‘diabetes drugs’ in combination with ‘primary hyperparathyroidism bone involvement’ or ‘primary hyperparathyroidism bone manifestation’ or primary hyperparathyroidism bone symptoms’ or ‘PHPT bone manifestation’ or ‘PHPT bone involvement’ or ‘PHPT bone symptoms’. The limits were set to only English-language articles and those involving human subjects. We included all case-control studies, case series and meta-analyses that were published from 2000 to date. Articles about normocalcemic PHPT were excluded.

Regarding our series, all blood samples were collected after overnight fasting and rest.

Serum total and urinary calcium and serum phosphate and creatinine levels were assayed by automated analysis using colorimetric and enzymatic methods, while ionized serum calcium was analyzed by a specific probe after pH correction.

The estimated glomerular filtration rate (eGFR) was calculated using the CKD-EPI formula (15): eGFR=141*min(SCr/k,1)α*max(SCr/k,1)-1.209*0.993*Age[*1.018 if women][*1.159 if black], where SCr represents serum creatinine (in mg/dL), k is 0.7 for women and 0.9 for men, α is −0.329 for women and −0.411 for men, min represents the minimum of SCr/k or 1, and max represents the maximum of SCr/k or 1.

Serum intact PTH concentrations were measured up to 2012 using a two-site immunochemiluminometric assay (Immulite 2000; DPC, Los Angeles, CA) with inter- and intra- assay variation coefficients of 6.3–8.8% and 4.2–5.7%, respectively. Thereafter, serum intact PTH concentrations were measured using a new second-generation immunochemiluminometric assay (Cobas e411, Roche Diagnostics) with inter- and intra-assay variation coefficients of 3.1–6.5% and 1.4–3.2%, respectively. Normal ranges are 20-65 pg/mL and 15-65 pg/mL, respectively.

Serum 25-hydroxy-vitamin D (25OHD) levels were measured by a radioimmunoassay (DIAsource 25OHVit. D3-Ria-CT Kit – DIAsource Immuno Assays S.A., Nivelles, Belgium), with a detection limit of 0.6 μg/L (1.5 nmol/L) and inter- and intra-assay variation coefficient of 5.3% and 4.7%, respectively. Our laboratory periodically conducts quality control tests on kit used with material provided by the manufacturer and is a member of the External Quality Assessment Scheme for the estimation of 25OHD, conducted by the QualiMedLab-CNR (Pisa, Italy), as a means of determining the accuracy of results. A level of less than 20 μg/L (50 nmol/L) was considered as the cut-off for vitamin D deficiency (VDD).

Total alkaline phosphatase activity (ALP) was evaluated using a colorimetric assay (Atellica CH, Siemens Healthineers) whose normal range is 33-98 U/L; the levels of bone-specific alkaline phosphatase isoenzyme activity (bALP) and osteocalcin were measured by chemiluminescent evaluation (CLIA, DiaSorin LIAISON XL). Normal ranges are 4.9-26.6 μg/L and 6.5-42.3 μg/L, respectively.

HbA1c was measured by HPLC (D-100 Bio-Rad); normal range in our laboratory is 4.3-5.7%.

BMD was measured at the lumbar spine (L2-L4), proximal femur, and distal third of the non-dominant radius using the same instrument (DXA QDR-4500, Hologic, Bedford, MA) throughout the study period. Minor upgrades to the BMD instrument, mainly in the reporting and duration of the procedure, did not significantly affect the results. Data were analyzed as absolute measurements (in grams per square centimeter) and reported as T scores.

All patients underwent standard renal ultrasound using a 2- to 5-MHz-wide band convex transducer. For a definitive diagnosis of stones, which enables patients to be classified as positive or negative for nephrolithiasis, radiologists looked for hyperechogenic spots that were more than 2 mm in diameter with a multiplanar evaluation of specific signs such as echogenicity, posterior acoustic shadowing, or a positive twinkle sign.

### Statistical Analysis

Variables were preliminarily tested for normal distribution with the Shapiro-Wilk’s W test and data were expressed as mean ± standard deviation (SD) when normally distributed, as median and interquartile range (IQR) when not normally distributed.

Continuous variables with non-normal and normal distribution were analyzed by Mann-Whitney U test and t-test for unpaired samples, respectively, as appropriate. Differences in categorical variables were analyzed by χ2 or Fisher’s test, as appropriate.

Linear regression analysis was used to evaluate the correlations between T2DM duration and glucose control and parameters related to PHPT.

Continuous variables with non-normal and normal distribution were analyzed among the three groups of patients with T2DM by Kruskal-Wallis and ANOVA, respectively.

The level of statistical significance was set at p ≤0.05. The calculations were performed using SPSS (IBM SPSS Statistics – Version 21).

## Results

Literature search did not retrieve any paper specifically evaluating the effect of diabetes therapy on PHPT manifestation.

As for our personal experience, data were analyzed from 472 consecutive patients diagnosed with sporadic PHPT in the considered period. A total of 55/472 (11.65%) patients had also T2DM.

The demographic, clinical and biochemical features of the whole series are summarized in **Table 1**, with a comparison between patients with and without T2DM.

**Table 1:** Demographic, clinical and biochemical data of PHPT patients with and without T2DM.

		With T2DM	Without T2DM	P*
**N**		55	417	
**Age (years)**		66.7 ± 9.8	60.5 ± 13.4	**0.001**
**Male sex**		29.1%	22.5%	0.309
**BMI (kg/m^2^)**		28.5 ± 5.8	24.8 ± 4.5	**<0.001**
**PTH (ng/L)**		116 [121]	130 [128]	0.091
**PHPT subtype**	**Symptomatic** **PHPT meeting surgical criteria** **Mild**	45.4% 36.4% 18.2%	51.3% 39.8% 8.9%	**0.005 ¥**
**Total serum calcium (mg/dL)**		11.4 ± 1	11.3 ± 1.1	0.121
**Ionized calcium (mmol/L)**		1.45 ± 0.1	1.46 ± 0.2	0.224
**Urinary calcium (mg/24h)**		232.1 ± 189.7	271 ± 160.6	0.158
**Serum phosphate (mg/dL)**		2.7 ± 0.5	2.6 ± 0.6	0.175
**eGFR (mL/min/1.73 m^2^)**		81.2 ± 26.7	85.9 ± 23.1	0.072
**25OHD (μg/L)**		25 ± 14.8	28.8 ± 19.3	0.125
**VDD**		27.3%	29.1%	0.358
**Lumbar T score**		-1.9 ± 1.7	-2.5 ± 1.4	**0.030**
**Femoral T score**		-1.4 ± 1.2	-2.1 ± 1.3	**0.006**
**Distal third radius T score**		-2.6 ± 1.7	-2.4 ± 1.6	0.148
**Osteoporosis at any site**		41.8%	50.8%	0.251
**Lumbar Z score**		-0.3 ± 1.7	-1.1 ± 1.4	**0.004**
**Femoral Z score**		-0.1 ± 1.2	-0.8 ± 1.1	**0.002**
**Distal third radius Z score**		-0.9 ± 1.7	-1.1 ± 1.4	0.576
**Osteitis fibrosa cystica**		18.2%	21.6%	**0.005**
**Osteocalcin (μg/L)**		31.8 ± 33.9	48.9 ± 40.5	**0.003**
**bALP (μg/L)**		14.02 ± 12	18.2 ± 17	0.09

*Bold points to statistically significant difference.

Continuous data with normal and not-normal distribution are expressed as mean ± SD and median [IQR], respectively.

¥ p value expresses the frequencies comparison between patients with and without T2DM.

No differences were found in gender distribution, serum levels of PTH, calcium, phosphate and 25OHD levels, and renal function. The prevalence of “mild asymptomatic” patients was significantly higher in the T2DM group. Patients with T2DM were significantly older, had higher BMI and a lower prevalence of OFC than non-diabetic patients. Lumbar and femoral T scores – but not forearm T scores – resulted significantly higher in T2DM patients.

The 55 diabetic patients with PHPT were grouped as follows according to the ongoing anti-diabetic therapy (this information was not available for 6 patients). Eleven out of 55 (20%) did not assume any drug for T2DM and were defined as group 1. T2DM was diagnosed concomitantly to PHPT in 6/11 patients of this group. Twelve out of 55 patients (21.8%) were treated with metformin alone and were classified as group 2. Twenty-six out of 55 (47.3%) were treated with insulin or secretagogues - sulfonylureas and glinides - whether or not taking metformin and were classified as group 3. The only patient treated with GLP-1 receptor agonist (in addition to insulin) was included in group 3.

**Table 2** shows the comparison between the PHPT patients with T2DM subdivided according to the ongoing antidiabetic drug at the moment of PHPT diagnosis.

**Table 2:** Data of PHPT patients with T2DM grouped according to antidiabetic treatment^§^.

		Group 1	Group 2	Group 3	P*
**N**		11	12	26	
**Age (years)**		66.3 ± 16	63.9 ± 5.5	68.5 ± 7.7	0.401
**Male sex**		27.3%	25%	30.8%	0.930
**BMI (kg/m^2^)**		28.2 ± 6	29.1 ± 5.3	28.2 ± 6	0.899
**HbA1c (%)**		6.7 ± 0.7	6.9 ± 0.9	7.7 ± 2.2	0.297
**T2DM duration (years)¥**		1.6 [0-7]	3.5 [0.5-11]	7.3 [0.5-38]	0.053
**PTH (ng/L)**		154 [89]	100 [124.75]	95.5 [151]	0.667
**PHPT subtype**	**Symptomatic** **PHPT meeting surgical criteria** **Mild**	54.5% 45.5% 0%	50% 25% 25%	46.2% 23% 30.8%	0.244
**Total serum calcium (mg/dL)**		11 ± 0.6	11.6 ± 1	11.2 ± 1.1	0.336
**Ionized calcium (mmol/L)**		1.41 ± 0.1	1.52 ± 0.2	1.42 ± 0.1	0.081
**Urinary calcium (mg/24h)**		287.5 [246.2]	228 [150]	173.4 [94.5]	0.157
**Serum phosphate (mg/dL)**		2.8 ± 0.4	2.6 ± 0.8	2.6 ± 0.4	0.626
**eGFR (mL/min/1.73 m^2^)**		88.8 ± 38.5	78.7 ± 18.4	77.5 ± 27.2	0.532
**25OHD (μg/L)**		33.6 ± 16.2	19 ± 9.9	23.7 ± 15.3	0.113
**VDD**		18.2%	41.7%	34.6%	0.143
**Lumbar T score**		-1.9 ± 1.7	-2 ± 2	-1.7 ± 1.8	0.922
**Femoral T score**		-1.4 ± 1	-1.7 ± 0.9	-1.5 ± 1.4	0.891
**Distal third radius T score**		-2.6 ± 1.7	-3.1 ± 1.5	-2.4 ± 1.8	0.657
**Osteoporosis at any site**		45.5%	58.3%	34.6%	0.382
**Lumbar Z score**		-0.2 ± 1.8	-0.4 ± 1.9	-0.2 ± 1.8	0.947
**Femoral Z score**		0.1 ± 0.9	-0.1 ± 0.9	- 0.2 ± 1.1	0.974
**Distal third radius Z score**		-0.7 ± 1.8	-1.2 ± 1.4	-0.9 ± 1.8	0.569
**Osteitis fibrosa cystica**		27.3%	16.7%	19.2%	0.807
**Osteocalcin (μg/L)**		32.8 ± 15.3	26.7 ± 10.1	36.5 ± 46.9	0.778
**bALP (μg/L)**		14 ± 11.8	17.8 ± 14.4	14.6 ± 12	0.547

§ group 1: no drugs for T2DM; group 2: metformin alone; group 3: insulin and/or secretagogues (± metformin).

*In bold significant differences at ANOVA.

¥ For clarity T2DM duration is reported as median and range.

Continuous data with normal and not-normal distribution are expressed as mean ± SD and median [IQR], respectively.

No differences were found among the 3 groups, with the exception of a nearly significant difference for T2DM duration.

No significant correlation were found between PHPT biochemical or radiological features and glycemic control or T2DM duration, with the exception of a significant inverse relation between the HbA1c and the forearm T score (p= 0.017, ß -0.402) and Z score (p= 0.011, ß -0.432).

## Discussion

The reported prevalence of T2DM in PHPT patients is approximately 8% (4), while that of PHPT in patients with T2DM is nearly 1%; in both cases values are three-fold higher than in general populations (16).

In PHPT, the increased frequency of glucose tolerance abnormalities has been associated with insulin resistance (3, 4, 17, 18).

While a relevant literature evaluated the coexistence of T2DM and PHPT (16, 19), only few data are available about T2DM and bone manifestations in PHPT, basically limited to bone turnover markers (17, 18). At this regard, osteocalcin, commonly measured as an index of bone formation, has also been proposed as a regulator of insulin sensitivity and a positive relationship between osteocalcin and glucose metabolism in PHPT has previously been reported (17).

On the other hand, no data are so far available about the impact of T2DM on PHPT bone classical manifestations, such as prevalence or expression of osteite fibrosa cystica.

Similarly, data about T2DM treatments and bone manifestations of PHPT are lacking.

For the first time our study specifically evaluated this topic and shows that the clinical presentation of PHPT is affected by the presence of T2DM. In fact, diabetic patients resulted more frequently “mild asymptomatic” than non-diabetic patients. Moreover, they showed a lower prevalence of radiological bone manifestations of PHPT, with higher lumbar and femoral T scores than patients without T2DM. On the other hand, the drugs used in the treatment of T2DM do not seem to affect the biochemical or clinical features of PHPT in our series.

The clinical profile of PHPT in Western countries has deeply changed over the last few decades, from a highly symptomatic disease, characterized by symptoms of hypercalcemia and kidney and bone involvement, to a largely asymptomatic disease (1).

Several observational studies in symptomatic PHPT reported an increased prevalence of metabolic abnormalities, especially IR and T2DM than in the general population (2–6). On the other hand, only limited controversial data exist about these features in patients with aPHPT.

Different mechanisms have been ascribed to the T2DM development in PHPT: hypercalcemia is associated with impaired insulin sensitivity and insufficient suppression of gluconeogenesis; moreover, PTH directly decrease glycolysis and stimulates hepatic glucose production (2).

At present, T2DM is not included among the criteria for surgery recommended by latest guidelines for aPHPT management (7), mainly due to the lack of data about glucose homeostasis improvement after PTX (2).

However, an increased fracture risk is a recognized complication of T2DM (8), in particular at the hip but also at the lumbar and radial level (20–22). The mechanisms underlying the effects of T2DM on skeletal health have been only partially elucidated. At this regard, obesity, poor muscle strength and increased risk of fall have consistently been observed in patients with T2DM (8, 20). Moreover, chronic hyperglycemia may alter calcium and vitamin D metabolism and cause the deposition of advanced glycosylation end-products in bone collagen, resulting in impaired bone quality (23, 24).

Previous studies showed that skeletal dynamics are impaired in T2DM with decreased osteoblast function, as documented by reduced biochemical markers of bone formation (8, 24). A difference in osteocalcin level between PHPT patients with or without T2DM has already been observed by our group in a previous study (18) and has been confirmed in the present extended and updated series.

Recent findings suggest that trabecular bone abnormalities may contribute to bone fragility in T2DM, despite normal – or even high – BMD (25, 26). It is interesting to note that, in our series, PHPT patients with T2DM showed significantly higher lumbar and femoral T scores than the remaining PHPT patients, while the BMD was equally impaired at the forearm. This finding strengthens the recommendation of the current guidelines to systematically perform DXA at three sites in all PHPT patients (7) and suggests that the omission of BMD measurement at the forearm may result in an underestimation of osteoporosis (27), in particular in PHPT patients with T2DM.

An optimal glycemic control is considered a relevant factor contributing to improvement of skeletal integrity in T2DM patients (24), even though data about a relationship between BMD and HbA1c in diabetic patients are inconclusive (28). In our series, the inverse correlation between HbA1c and T score reached the statistical significance only at the forearm, suggesting a possible amplification of the typical cortical damage due to PHPT.

Actually, also antidiabetic drugs may have both direct and indirect effects on bone health and fracture risk (8, 20). Data about metformin are conflicting, reporting a positive or neutral effect on BMD and fracture risk in different, large cohorts (29, 30). TZD activate peroxisome proliferator-activated receptors (PPARs), with greatest specificity for PPARγ, resulting in increased adipogenesis and impaired osteoblastogenesis. Their long-term use is associated with a greatly increased fracture risk, both in men and women (8, 9). A meta-analysis of trials assessing BMD changes reported greater bone loss at the lumbar and femoral sites in women treated with TZD compared with other antidiabetic drugs (31). These drugs are thus not recommended in postmenopausal women with low bone density or other risk factors for fracture.

Data about skeletal safety of sulfonylureas (SU) are scarce. A systematic review on this topic reported that the risk of fall and fractures in SU users may be underestimated, mostly because no clinical trial included fracture as a primary endpoint and often older adults were excluded (32). However, whilst the mechanisms of SU on BMD and on bone metabolism are still unknown, hypoglycemia seems to reasonably explain at least in part the SU-induced fractures (9).

Several studies reported that endogenous insulin induces anabolic effects on the bone, which includes the regulation of bone cell proliferation and apoptosis, while the effect of exogenous insulin remains to be fully elucidated (33, 34). It should be taken into account that patients with T2DM treated with insulin usually have a long history of DM with an increased risk of DM complication, such as retinopathy, peripheral neuropathy, and balance impairment, that may affect the occurrence of fall and bone fractures (27, 35).

The effect on fracture risk of the incretin-based drugs used in the treatment of T2DM has not been fully established (8, 24). Incretins seems to play a regulatory role in the complex regulation of bone turnover in response to feeding, enhancing bone formation in response to nutritional intake (36). In experimental studies, subcutaneous glucagon-like peptide (GLP) -2 daily administration resulted in a dose-dependent increase in hip BMD in postmenopausal women after 4 months of treatment (37). A meta-analysis of randomized controlled trials reported different effects of GLP-1 analogues on fracture risk, with a protective effect of liraglutide (37).

Clinical evidence is lacking also for DPP-4 inhibitors; the potential neutral or beneficial effects of these agents shown in preclinical studies and clinical trials need to be confirmed in clinical series (2, 12, 25). From a clinical perspective, on the basis of the available data, incretin-based therapy seems to be an ideal treatment to reach glycemic control in diabetic patients with high risk of fracture, also due to their low risk of hypoglycemia.

An influence of SGLT-2 inhibitors on bone metabolism has been reported. These drugs reduce the absorption of glucose and sodium in the renal proximal tubule, where the sodium-dependent phosphate co-transporters are expressed. This leads to an increased reabsorption of phosphate, which triggers FGF-23 secretion and consequently causes a decline in 1,25-dihydroxyvitamin D levels, with a secondary PTH elevation (8–10). SGLT-2 inhibitors may thus have adverse skeletal effects by altering calcium and phosphate homeostasis that might lead to decline in BMD and increased risk of bone fracture. However, at present, there is uncertainty about their effects on fracture risk. Canagliflozin has been previously associated with increased fracture risk compared to other drugs used for diabetic treatment (10). In contrast, a meta-analysis on patients with T2DM treated with SGLT-2 inhibitors compared with placebo did not observe a significantly increased risk of fracture, either if molecules of this class were evaluated individually or as a class effect. The results were limited by short duration of follow-up and low incidence of the event of interest (38). As for BMD, a study showed no significant changes after 50 weeks of dapagliflozin treatment (39), while a decrease at the lumbar and femoral sites was reported after a 52-week canagliflozin treatment (40). To date, factors contributing to fracture risk should be considered before prescribing SGLT-2 inhibitors.

To the best of our knowledge, no data are still available about the relationship between drugs used for T2DM treatment and classical bone manifestation of PHPT, thus our study is the first addressing this topic. We found no differences in the classical bone manifestations of PHPT among the different groups of antidiabetic treatments. It is worth noting that our series includes patients evaluated over a large time period, when SU and insulin were extensively prescribed. As for the most recently drugs approved for the treatment of T2DM, in our series only one patient was treated with a GLP-1 receptor agonist and none was taking a SGLT-2 inhibitor. Since no patients were taking drugs clearly associated with a greatly increased fracture risk, such as TZD, this aspect is not fully evaluable in our series. Another limitation of our study is the lack of results about the hard endpoint of fractures, but this is intrinsically linked to the retrospective cross-sectional design of this study.

The prevalence of PHPT and T2DM has widely increased in the last few decades and their concomitance is supposed to become more and more frequent, in particular in older adults. Mainly in these patients the simultaneous occurrence of conditions that increase the risk of fragility fractures may impair quality of life and increase institutionalization rate and mortality (41).

In patients with concomitant PHPT and T2DM, the BMD measurement at the forearm may be particularly relevant for a proper clinical work-up. Moreover, the avoidance of anti-diabetic medication with adverse skeletal effect needs to be carefully considered, in particular when additional factors predisposing to fracture risk, as the postmenopausal state, are present. Further studies are needed to clarify which class of antidiabetic drugs is the most suitable in PHPT patients, even though at present metformin and incretin-based drugs are likely the drugs of choice.

## Author Contributions

EC and GB conceived of the presented idea. EC and RA developed the theory and performed the computations. AB and VB verified the analytical methods. FT and GB supervised the findings of this work. All authors discussed the results and contributed to the final manuscript. All authors contributed to the article and approved the submitted version.

## Conflict of Interest

The authors declare that the research was conducted in the absence of any commercial or financial relationships that could be construed as a potential conflict of interest.

## Abbreviations

25OHD, 25-hydroxy-vitamin D; aPHPT, asymptomatic PHPT; bALP, bone-specific alkaline phosphatase isoenzyme activity; BMD, bone mineral density; BMI, body mass index; CV, cardiovascular; DM, diabetes mellitus; DPP-4, dipeptidyl-peptidase 4; DXA, dual X-ray absorptiometry; eGFR, estimated glomerular filtration rate; FGF, fibroblast growth factor; GLP, glucagon-like peptide; HPLC, high pressure liquid chromatography; IQR, interquartile range; IR, insulin resistance; MEN, multiple endocrine neoplasms; OFC, osteitis fibrosa cystica; PHPT, primary hyperparathyroidism; PPAR, peroxisome proliferator-activated receptor; PTH, parathyroid hormone; PTX, parathyroidectomy; SD, standard deviation; SGLT-2, sodium-glucose transporter 2; SU, sulfonylureas; T2DM, type 2 diabetes mellitus; TZD, thiazolidinediones; VDD, vitamin D deficiency.
